# Two new species in the *Matelea stenopetala* complex (Apocynaceae, Asclepiadoideae) from the Guiana Shield and Amazonian Brazil

**DOI:** 10.3897/phytokeys.17.3485

**Published:** 2012-09-26

**Authors:** Alexander Krings, Gilberto Morillo

**Affiliations:** 1 Herbarium, Department of Plant Biology, North Carolina State University, Raleigh, NC 27695-7612, USA; 2 Herbario Carlos Liscano, Facultad de Ciencias Forestales y Ambientales, Universidad de Los Andes, Mérida 5101-A, Venezuela

**Keywords:** Gonolobinae, *Matelea*, Neotropics, twining vines, taxonomy

## Abstract

Two new species in the *Matelea stenopetala* complex (Apocynaceae, Asclepiadoideae) are described from the Guiana Shield and Amazonian Brazil: *Matelea brevistipitata* Krings & Morillo, **sp. nov.** and *Matelea trichopedicellata*Krings & Morillo**, sp. nov.** The new species belong to a small group of adaxially-pubescent-flowered taxa within the complex, including *Matelea hildegardiana* and *Matelea pakaraimensis*. The new species are described and a dichotomous key is provided.

## Introduction

The *Matelea stenopetala* Sandwith complex (Apocynaceae, Asclepiadoideae, Gonolobinae) in northern South America includes about ten species of vines (Morillo 1988, 1991; Farinaccio and Stevens 2009; Krings 2011). Members of the complex are recognized by leaves membranous, often more or less elliptic or oblong and relatively large (to ca. 17 x 8 cm), leaf bases frequently narrow and cuneate or acute, flowers small (corolla lobes usually ≤ 4–5 mm long [to 5.8 mm in *Matelea cayennensis* Morillo]), typically green to greenish-yellow, reticulate but not ocellate, and gynostegial coronas not elaborate, the staminal corona segments (Cs) usually appearing as ridges emanating from the central stipe and the interstaminal corona segments (Ci) appearing as sinuses in between the Cs. The morphology of the flowers is strikingly similar to *Matelea palustris* Aubl. [type of *Matelea* Aubl.], the affinity to which has been pointed out by Sandwith (1931: his protologue of *Matelea stenopetala*), as well as by Farinaccio and Stevens (2009). On-going work for various regional initiatives, including the Flora of the Guianas and the Biological Diversity of the Guiana Shield, has resulted in the discovery of two new species referable to the *Matelea stenopetala* complex. Both new species exhibit corolla lobes adaxially pubescent, a character state shared in the complex only by *Matelea hildegardiana* Morillo (Venezuela), *Matelea pakaraimensis* Krings (Guyana), and, very rarely, *Matelea stenopetala* (northern South America). The new species are described and distinguished below. Corona morphological terminology follows Liede and Kunze (1993), although it is recognized that additional work may be needed to clarify issues of family-wide homology (see Endress and Bruyns 2000, Livshultz 2003). Shorthand abbreviations for corona morphology following Liede and Kunze (1993) are as follows: Ca = annular corona of corolline derivation (faucal annulus); Ci = interstaminal corona; C(is) = fused interstaminal and staminal corona; Cs = staminal corona.

## Data resources

The data underpinning the analysis reported in this paper are deposited at GBIF, the Global Biodiversity Information Facility, http://ipt.pensoft.net/ipt/resource.do?r=two_new_species_in_the_matelea_stenopetala_complex_from_the_guiana_shield_and_amazonian_brazil

## Taxonomic treatment

### Key to the adaxially-pubescent-flowered members of the *Matelea stenopetala* complex

**Table:** 

1	Abaxial corolla surface conspicuously pubescent, trichomes predominantly eglandular, glandular capitate trichomes absent or sparse and inconspicuous	2
–	Abaxial corolla surface glabrous or minutely and indistinctly pubescent, trichomes primarily glandular capitate, eglandular trichomes absent or rare	3
2	Leaf bases broadly cuneate to rounded; pedicels with only glandular capitate trichomes ubiquitous, eglandular trichomes not ubiquitous, instead in two lines, ca. 0.3 mm long; corolla rose, lobes 4.1–4.2 mm long; Figs 1A, 2A, 3A, 4B, 6	*Matelea trichopedicellata*
–	Leaf bases attenuate to narrowly cuneate; pedicels with both glandular capitate and eglandular trichomes ubiquitous, eglandular trichomes to 0.07 mm long; corolla green, lobes 4.9–5.5 mm long; Figs 1B, 2B, 3B	*Matelea hildegardiana*
3	Corolla purplish; C(is) not distinctly raised off the corolla surface by a subtending stipe; Figs 1C, 2C	*Matelea pakaraimensis*
–	Corolla green, greenish-yellow, yellow, or cream; C(is) distinctly raised off the corolla surface by a subtending stipe	4
4	Open flowers per inflorescence usually 1(–2); peduncles 1.9–10.0 (–16.0) mm [avg. 7 mm]; calyx green, sometimes purplish; stipe subtending C(is) 0.12–0.22 mm [avg. 0.17 mm] tall; Figs 1D, 2D, 3C, 4A, 5 *Matelea brevistipitata*
–	Open flowers per inflorescence usually (2–) 3–4 (–5); peduncles (4–) 8–26 mm [avg. 15 mm]; calyx frequently purple, sometimes black or brown; stipe subtending C(is) 0.3–0.55 mm tall [avg. 0.37 mm]; Figs 1E & F, 2 E & F, 3D	*Matelea stenopetala*

### Matelea brevistipitata

Krings & Morillo sp. nov.

urn:lsid:ipni.org:names:77122392-1

http://species-id.net/wiki/Matelea_brevistipitata

**Figure 1.**
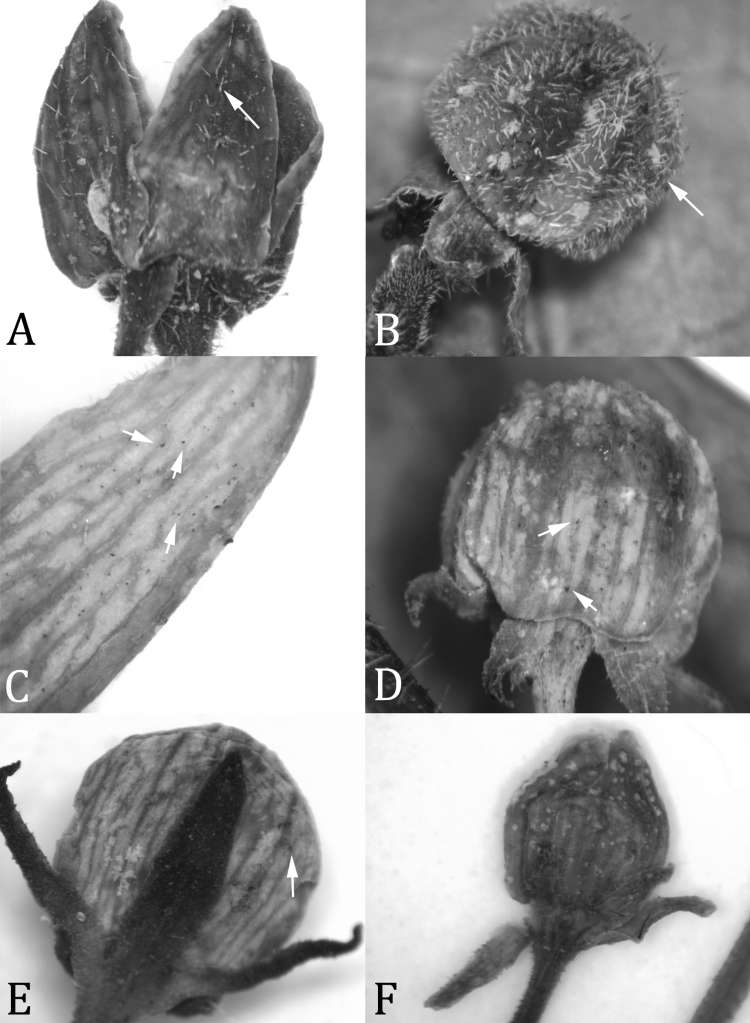
Abaxial corolla surface in the adaxially-pubescent-flowered members of the *Matelea stenopetala* complex. **A** *Matelea trichopedicellata* (based on *Daly et al. 1619*, US) **B** *Matelea hildegardiana* (based on *Liesner 23469*, U) **C** *Matelea pakaraimensis* (based on *Mutchnik 122*, US) **D** *Matelea brevistipitata* (based on *Boom 9318*, VEN) **E** *Matelea stenopetala* (based on *Ek et al. 881*, U) **F** *Matelea stenopetala* (based on *Mori et al. 24547*, US); Note difference in pubescence type between **A–B** (eglandular trichomes shown at arrows) and **C–F **(glandular capitate trichomes shown at arrows).

Figures 1D 2D 3C 4A 5

#### Latin.

*A new species in the Matelea stenopetala complex, most similar to M. stenopetala, but differing in part by inflorescences with fewer flowers open at a time (usually 1(–2) vs. (2–) 3–4 (–5) in M. stenopetala), shorter peduncles (avg. 7 mm vs. avg. 15 mm in M. stenopetala), and stipe subtending the C(is) 0.12–0.22 mm [avg. 0.17 mm] tall (vs. 0.3–0.55 mm tall [avg. 0.37 mm] in M. stenopetala]*.

#### Type.

**VENEZUELA**. BOLÍVAR: Cerro Guaiquinima, Base Camp (Camp 7) along the Río Canapo, tropical lowland evergreen forest, near river, 5°N, 63°W, 310 m, 3 Feb 1990 (fl), *B. Boom 9318* (Holotype: VEN!; Isotype: NY!).

#### Description.

*Slender, woody vine*. *Stems* glabrescent to moderately pubescent, pubescence in two lines, eglandular trichomes retrorse or spreading, of different size classes, largest ca. 0.5 mm long, smallest ca. 0.08 mm long, glandular capitate trichomes sparse to moderately dense, spreading, ca. 0.1 mm long. *Leaf* blades lanceolate, ovate, elliptic, oblong, obovate, or oblanceolate, 5.2–11.5 × 1.1–5.2 cm, with 5–7 pairs of lateral veins, adaxial surface glabrous, midvein glabrous to moderately pubescent, eglandular trichomes spreading, ca. 0.3 mm long, glandular capitate trichomes spreading, ca. 0.1 mm long, abaxial surface glabrous, midvein glabrous or sparsely pubescent, eglandular trichomes spreading, ca. 0.3 mm long, glandular capitate trichomes spreading, ca. 0.1 mm long, apices acuminate, bases cuneate to rounded, margins entire, colleters 3–4, lanceolate, 0.4–0.6 mm long; petioles 0.7–3.0 cm long, moderately pubescent, eglandular trichomes ubiquitous but most dense along adaxial ridge, spreading to antrorse, 0.4–0.5 mm long, glandular capitate trichomes ubiquitous, spreading, ca. 0.05 mm long. *Inflorescence* racemiform, 3–5-flowered (1(–2) flowers open at a time); peduncles 1.9–10 (–16) mm, sparsely to moderately pubescent, pubescence ubiquitous, eglandular trichomes rare, spreading or antrorse, ca. 0.2–0.25 mm long, glandular capitate trichomes spreading, ca. 0.1 mm long; pedicels 6.3–15.0 mm long, sparsely to moderately pubescent, pubescence ubiquitous, eglandular trichomes rare, spreading or antrorse, ca. 0.1 mm long, glandular capitate trichomes spreading, ca. 0.05 mm long. *Calyx* lobes linear to lanceolate, 1.1–2.0 × 0.25–0.45 mm, strongly reflexed, adaxial surface glabrous, abaxial surface sparsely to moderately pubescent, eglandular trichomes spreading or antrorse, ca. 0.3 mm, glandular capitate trichomes spreading, 0.03–0.05 mm long, apices obtuse, margins entire; colleters 1 per sinus, lanceolate, ca. 0.2 mm tall. *Corolla* green, greenish-yellow, or greenish-white (fide collectoris), subcampanulate at base, tube ca. 0.5–0.7 × 0.8–0.9 mm, lobes imbricate in bud, lanceolate to oblong, apparently spreading, 3.0–4.1 × 1.7–1.9 (–2.2) mm, not ocellate, adaxial surface pubescent, eglandular trichomes spreading, ca. 0.05–0.1 mm long, glandular-capitate trichomes absent, abaxial surface sparsely pubescent, eglandular trichomes rare, ca. 0.07 mm long, glandular capitate trichomes spreading, ca. 0.03 mm long, apices obtuse, margins entire. *Faucal annulus* (corolline corona or Ca) absent. *Gynostegial corona* of fused staminal (Cs) and interstaminal (Ci) parts, C(is) subtended by a short stipe, stipe 0.12–0.22 mm tall, Cs rising to meet the lower portion of the anther, rising segment at a distinctly acute angle relative to the base, 0.6–0.66 mm tall, margin entire, base somewhat swollen, entire or crenulate-lobate, ligule an apical ridge, not free, Ci unlobed, not ligulate. *Style-head* 1.2–1.4 (–1.7) mm diam, stipe 0.9–1.1 mm tall (incl. section subtending the C(is)), terminal style-head appendage absent. *Pollinarium*: corpuscula ca. 0.12–0.17 mm long, caudicles present, pollinia oblong, 0.36–0.4 × 0.15–0.25 mm. *Follicles* unknown. *Seeds* unknown.

**Figure 2.**
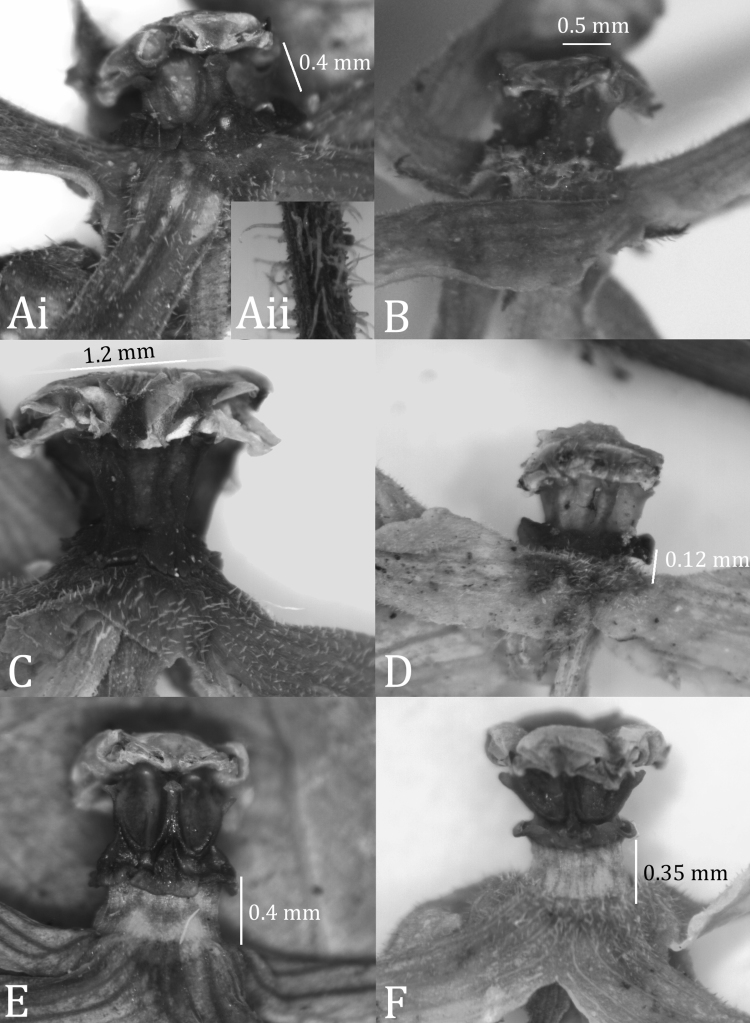
Gynostegial variation in the adaxially-pubescent-flowered members of the *Matelea stenopetala* complex. **A** *Matelea trichopedicellata*: i, flower, ii, coarsely pubescent pedicel, trichomes ca. 0.3 mm long (based on *Daly et al. 1619*, US) **B** *Matelea hildegardiana* (based on *Davidse 4903*, U) **C** *Matelea pakaraimensis* (based on *Mutchnik 122*, US) **D** *Matelea brevistipitata* (based on *Boom 9318*, VEN) **E** *Matelea stenopetala* (based on *Ek et al. 881*, U) **F** *Matelea stenopetala* (based on *Mori et al. 24547*, US). Note coronas at base of gynostegial columns sessile in A–C and raised off the surface of the corolla by a subtending stipe in **D–F**.

**Figure 3.**
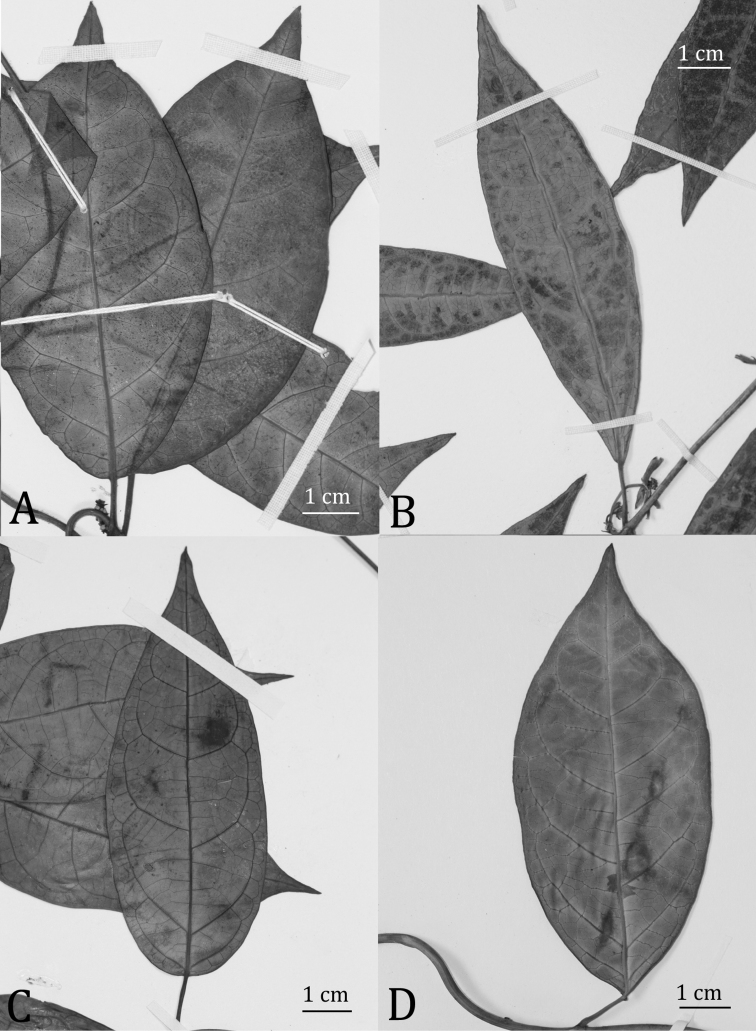
Foliar variation in some adaxially-pubescent-flowered members of the *Matelea stenopetala* complex. **A** *Matelea trichopedicellata* (based on *Daly et al. 1619*, US) **B** *Matelea hildegardiana* (based on *Davidse 4903*, US) **C** *Matelea brevistipitata* (based on *Boom 9318*, VEN) **D** *Matelea stenopetala* (based on *Ek et al. 881*, U).

**Figure 4.**
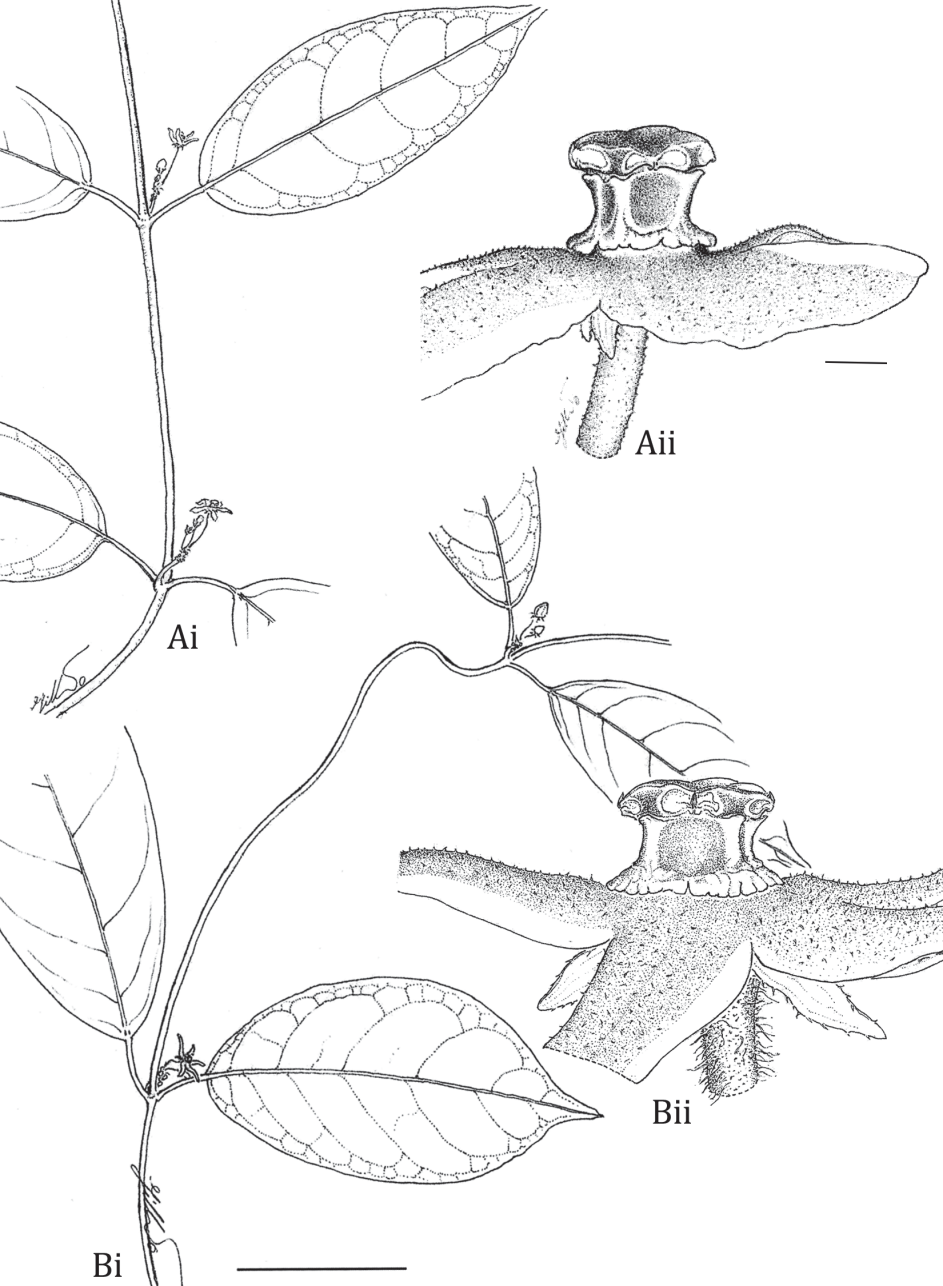
Illustration of the leaves, inflorescences, and flowers of *Matelea brevistipitata* and *Matelea trichopedicellata*. **A** *Matelea brevistipitata* (based on *B. Stergios 12315*, NY) **B** *Matelea trichopedicellata* (based on *Daly et al. 1619*, US). Scale bars are 4 cm (Ai & Bi) and 0.4 mm (Aii & Bii).

**Figure 5.**
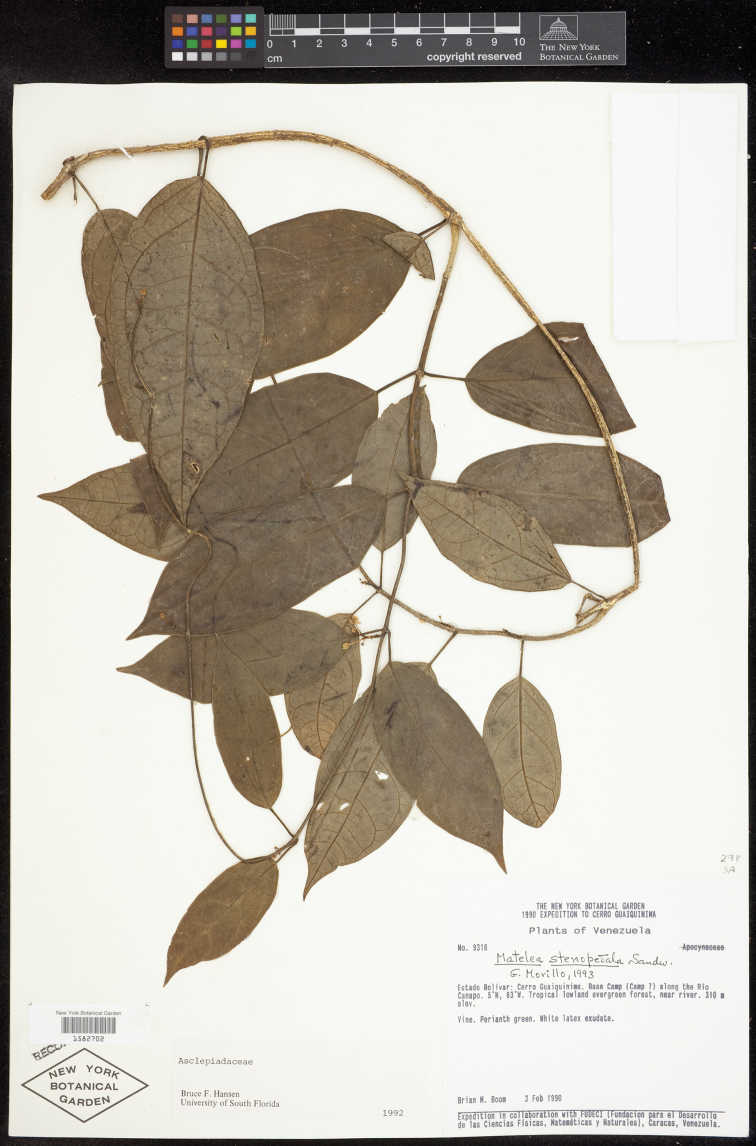
Isotype of *Matelea brevistipitata* (*Boom 9318*, NY). Courtesy of the New York Botanical Garden.

#### Distribution.

*Matelea brevistipitata* isapparently endemic to the Guiana Shield in the Venezuelan states of Amazonas and Bolívar. It is very likely also present in the portion of Amazonian Brazil bordering those states. The distributions of *Matelea brevistipitata* and *Matelea stenopetala* appear to be largely parapatric. Both species occur in Bolívar (Venezuela), but this represents the easternmost edge of the range of *Matelea brevistipitata* and the westernmost edge of the range of *Matelea stenopetala*.

#### Ecology.

Known from swamp and riverine forests at low elevations, to 500 m.

#### Phenology.

Collected in flower in April, May, October, and November.

#### Etymology.

The specific epithet refers to the short stipe subtending the C(is).

#### Conservation status.

Currently, very little is known regarding the status of this species.

#### Discussion.

At a glance, *Matelea brevistipitata* can be distinguished from *Matelea stenopetala* by the few-flowered inflorescences, suggesting divergence in reproductive biology. *Matelea stenopetala* usually displays inflorescences with 3–4 open flowers, whereas *Matelea brevistipitata* displays inflorescences with only one open flower (rarely two) at a time. The floral display itself also differs between the two species. Although both exhibit calyx lobes strongly reflexed at anthesis, the lobes of *Matelea stenopetala* tend to be purple (sometimes also black or brown), presenting a striking contrast to the light colored corolla. In *Matelea brevistipitata*, in contrast, the calyx is usually green (frequently drying an olive green). While the adaxial corolla surface is pubescent in *Matelea brevistipitata*, it is only very rarely so in *Matelea stenopetala*. In fact, the vast majority of specimens of *Matelea stenopetala* seen exhibit corollas adaxially glabrous. It is worth noting that the rare exception (i.e., *Mori 24547*, US) otherwise exhibits character states typical of *Matelea stenopetala*, including long peduncles (13–16 mm), inflorescences with up to five flowers open at a time, and long stipes subtending the C(is) (0.35 mm; Fig. 2F).

#### Specimens examined.

Venezuela: **Amazonas**. Camino entre caño Gruya y el pueblo de Gruya, bosque higrófilo macrotérmico ligeramente alterado en la margen del río Orinoco, 100 m, 8 Apr 1978 (fl), *G. Morillo 7382* (VEN); Dept. Atabapo, Salto Yureba, Caño Yureba, Bajo Ventuari, 4°3'N, 66°1'W, 120–150 m, 24 Oct–4 Nov 1981 (fl), *F. Delascio & F. Guánchez 10968* (MER,VEN); Dept. Atures, carretera Pto. Ayachucho-Samariapo, selva húmeda caliente, 13 Jan 1982 (fl), *B. Stergios 3183* (VEN); Dept. Río Negro, between Río Marawinuma at base of Neblina (0°50'N, 66°9'W) and Río Baría, in swamp forest, a heavily overgrown series of small channels with black water, 8 May 1984 (fl), *W.W. Thomas, A. Gentry & B. Stein 3401* (VEN, NY). **Bolívar**. Dept. Roscio, El Abismo, al norte del río Icabarú, bosque húmedo macrotérmico, 500 m, Oct 1985 (fl), *F. Delascio 12503* (VEN); Expedición Proyecto I.R.N.R.S. a la cuenca alta del Río Caura (Hoja NB-20-14), selvas ribereñas del Caño El Pavo hasta arriba de la boca del Caño Maravene, 4°16'N, 64°9'W, 13–14 Apr 1988 (fl), *B. Stergios 12315* (NY, US, VEN); Dist. Piar, Río Aparamán, at rapids of Yuray-merú, tributary of Río Acanan, SW base of Amaruay-tepui, E of Auyan-tepui, W of Aparaman-tepui, small tributary or river, densely forested with trees 20–30 m high, 5°55'N, 62°15'W, 500 m, 21 Apr 1986 (fl), *B. Holst & R. Liesner 2661* (VEN, MO).

### Matelea trichopedicellata

Krings & Morillo sp. nov.

urn:lsid:ipni.org:names:77122393-1

http://species-id.net/wiki/Matelea_trichopedicellata

Figures 1A 2A 3A 4B 6

#### Latin.

*A new species in the Matelea stenopetala complex, most similar to M. hildegardiana, but differing in part by leaf bases broadly cuneate to rounded (vs. attenuate to narrowly cuneate in M. hildegardiana), pedicels coarsely pubescent, with only glandular capitate trichomes ubiquitous, eglandular trichomes not ubiquitous, rather in two lines, ca. 0.3 mm long (vs. both glandular capitate and eglandular trichomes ubiquitous, eglandular trichomes to 0.07 mm long in M. hildegardiana), and corollas rose, lobes 4.1–4.2 mm long (vs. corolla green, lobes 4.9–5.5 mm long in M. hildegardiana)*.

#### Type.

**BRAZIL**. PARÁ: Rio Tocantins near Igarapé Cajazeirinha, approx. 30 km N of Itupiranga, 4°1'S, 49°21W, 1 Dec 1981 (fl & fr), *D.C. Daly, R. Callejas, M.G. da Silva, E.L. Taylor, C. Rosario, & M.R. dos Santos 1619* (Holotype: NY!; Isotype: US!, MG, n.v., MO, n.v.).

#### Description.

*Slender, woody vine*. *Stems* moderately to densely pubescent, pubescence in two lines, eglandular trichomes coarse, retrorse, ca. 0. 4 mm long, glandular capitate trichomes sparse, spreading, ca. 0.05 mm long. *Leaf* blades narrowly to broadly elliptic, 5.7–7.8 × 2.0–4.5 cm, with 5–8 pairs of lateral veins, adaxial surface glabrous, midvein glabrous or pubescent, eglandular trichomes antrorse or spreading, ca. 0.3 mm long, glandular capitate trichomes spreading, ca. 0.05–0.07 mm long, abaxial surface glabrous, midvein glabrous or pubescent, eglandular trichomes antrorse or spreading, ca. 0.3 mm long, glandular capitate trichomes spreading, ca. 0.05-0.07 mm long, apices acuminate, bases broadly cuneate to rounded, margins entire, colleters 2–4, lanceolate, 0.3–0.4 mm long; petioles 0.9–1.3 cm long, moderately pubescent, pubescence ubiquitous, eglandular trichomes mostly restricted to the adaxial ridge, spreading to antrorse-spreading, ca. 0.4 mm long, glandular capitate trichomes spreading, 0.05–0.07 mm long. *Inflorescence* racemiform, apparently 1–2-flowered (1 flower open at a time); peduncles to 2.4 mm long, moderately pubescent, pubescence ubiquitous, eglandular trichomes antrorse or spreading, 0.2–0.25 mm long, glandular capitate trichomes spreading, 0.05–0.07 mm long; pedicels 4.4–5.2 mm long, moderately to densely pubescent, eglandular trichomes in two lines, spreading, ca. 0.3 mm long, glandular capitate trichomes ubiquitous, spreading, ca. 0.05–0.07 mm long. *Calyx* lobes lanceolate, reflexed or spreading, 1.4–1.7 × 0.4–0.6 mm, adaxial surface glabrous, abaxial surface moderately to densely pubescent, eglandular trichomes antrorse or spreading, ca. 0.18 mm long, glandular capitate trichomes absent or very sparse, spreading when present, ca. 0.06 mm long, apices obtuse, margins entire; colleters 1 per sinus, lanceolate, ca. 0.18 mm tall. *Corolla* rose (fide collectoris), subcampanulate at base, tube ca. 0.9 × 0.9 mm, lobes imbricate in bud, linear-lanceolate to oblong, spreading, 4.1–4.2 × 1.9–2.0 mm, not ocellate, marginally or laterally recurved, adaxial surface pubescent, eglandular trichomes spreading, ca. 0.06–0.08 mm long, glandular-capitate trichomes absent, abaxial surface moderately pubescent, eglandular trichomes antrorse, 0.15–0.18 mm long, glandular capitate trichomes absent or very sparse, spreading when present, 0.03–0.04 mm long, apices obtuse, margins entire. *Faucal annulus* (corolline corona or Ca) absent. *Gynostegial corona* of fused staminal (Cs) and interstaminal (Ci) parts, C(is) on the surface of the corolla lobes, not subtended by a stipe, margin crenulate-lobate, Cs rising to meet the lower portion of the anther, rising segment more or less perpendicular relative to the base, ca. 0.4 mm tall, margin entire, base somewhat swollen, crenulate-lobate, ligule an apical ridge, not free, Ci unlobed, not ligulate. *Style-head* 1.6–1.7 mm diam, stipe 0.45–0.65 mm tall, terminal style-head appendage absent. *Pollinarium*: corpuscula ca. 0.18 mm long, caudicles present, pollinia narrowly ovoid, ca. 0.45 × 0.2 mm. *Follicles* ovoid-fusiform (imm), ca. 6 x 1.3 cm. *Seeds* unknown.

**Figure 6.**
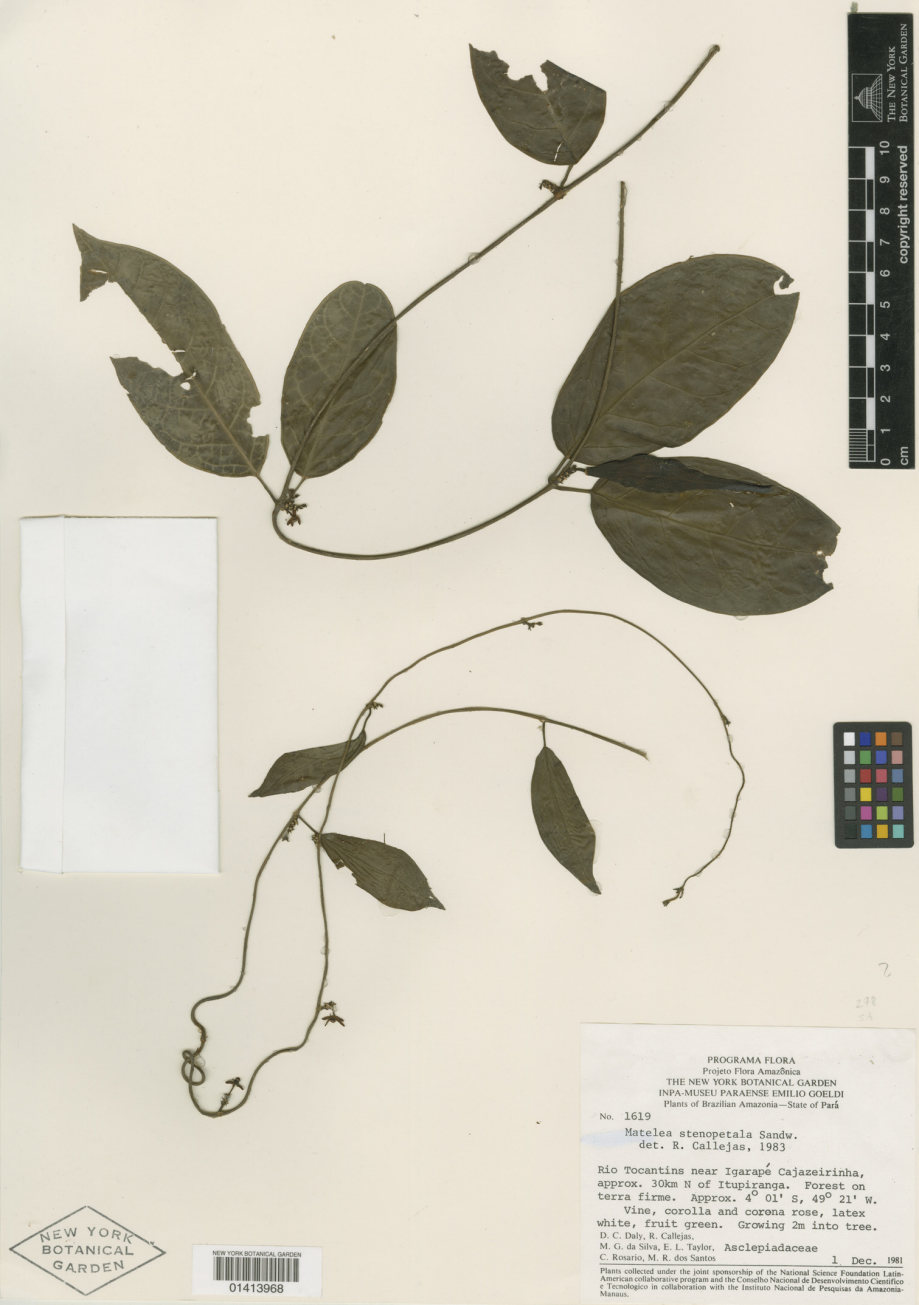
Holotype of *Matelea trichopedicellata* (*Daly et al. 1619*, NY). Courtesy of the New York Botanical Garden.

#### Distribution.

Known only from the type, collected in the Brazilian Amazon, near Igarapé Cajazeirinha.

#### Ecology.

Currently known only from terra firme forest.

#### Phenology.

Collected in flower and fruit (imm) in December.

#### Etymology.

The specific epithet refers to the conspicuously pubescent pedicels.

#### Conservation status.

Currently, very little is known regarding the status of this species.

#### Discussion.

*Matelea trichopedicellata* shares with *Matelea hildegardiana* (apparently endemic to the Gran Sabana, Bolívar, Venezuela), pubescent abaxial and adaxial corolla surfaces, but differs from it in part by leaf bases broadly cuneate to rounded and corolla lobes rose, 4.1–4.2 mm long (leaf bases attenuate to narrowly cuneate, and corolla lobes green, 4.9–5.5 mm long in *Matelea hildegardiana*). *Matelea trichopedicellata* and *Matelea hildegardiana* both exhibit eglandular trichomes on the abaxial corolla lobe surface in which the lowest cell is usually filled with a green or dark substance. The upper cells of the trichomes are usually translucent.

## Supplementary Material

XML Treatment for Matelea brevistipitata

XML Treatment for Matelea trichopedicellata
